# Applying the Public Health Nursing Intervention Wheel to a Paternal Perinatal Mental Health Case Study

**DOI:** 10.1111/phn.70099

**Published:** 2026-02-24

**Authors:** Lloyd Frank Philpott, Helen Mulcahy

**Affiliations:** ^1^ School of Nursing and Midwifery University College Cork Cork Ireland

**Keywords:** case study, education, interventions, paternal perinatal mental health, public health nursing intervention wheel, public health nursing

## Abstract

**Background:**

Adverse paternal perinatal mental health is recognized as a serious public health concern due to the negative implications for fathers, their families, and wider society. However, in the Irish healthcare system there is no evidence that the assessment and management of paternal perinatal mental health is part of current public health nursing practice.

**Case presentation:**

The authors designed a hypothetical case study and used it with the public health nursing intervention wheel to analyze and illustrate how public health nursing practice is more likely to focus on maternal and infant health outcomes.

**Findings:**

The case illustrated that there are opportunities to integrate applicable interventions such as assessment, screening, consultation, counseling, health teaching, referral, and follow‐up at paternal and family level, as well as outreach, advocacy, and social marketing at community level. Evidence from the research‐based literature is used to discuss how these interventions can result in beneficial outcomes for fathers, families and population health.

**Conclusion:**

Furthermore, it illustrates how case studies can enhance teaching in relation to public health nursing education.

## 1 Introduction

### 1.1 Paternal Perinatal Mental Health

The World Health Organization's (WHO) proposition that there can be “no health without mental health” has been endorsed by governments and organizations globally (Clark et al. 2024). As a result, there has been increased awareness regarding the importance of mental health in general, and for perinatal mothers; however, the mental health of fathers during the perinatal period is only now beginning to be recognized in research and, to a much lesser extent, in clinical practice (Fisher et al. 2021). The research undertaken has reported that positive mental health for new fathers is critical to ensure beneficial outcomes for their infant, partner, and wider society (Philpott 2023).

To date, systematic reviews of paternal perinatal stress, anxiety, and depression have reported prevalence rates of 6%–69%, 2%–51%, and 8%, respectively (Philpott et al. 2017, 2019; Cameron et al. 2016). The reported rates of adverse paternal perinatal mental health outcomes represent a significant public health concern; however, it has been suggested that the true prevalence may well be underestimated as men tend to under‐report mental health symptoms, leading to underdiagnosis (Philpott et al. 2020). Research examining paternal perinatal mental health has primarily focused on depression, with less attention given to stress and anxiety. More recently, there is evidence that stress, anxiety, and depression frequently co‐exist, thus, a focus on depression does not accurately represent the substantive risk to paternal mental health in the perinatal period (Philpott et al. 2022).

### 1.2 Public Health Nurses and Perinatal Care Interventions

Support interventions received in the perinatal period are vital for women in their transition to motherhood (Giltenane et al. 2021). The Public Health Nurse (PHN) is in a unique position to provide the necessary support to ensure a smooth transition (Walsh et al. 2023). In the Irish healthcare system, PHNs work with families from the first visit in the early postnatal period following birth, until the child reaches school‐going age (Walsh et al. 2023). As a result, every family with a child under the age of 5 will have routine contact with a PHN. Through these contacts, PHNs have the capacity to support new parents in their transition to parenthood, promote child development, improve child health outcomes, and ensure families at risk are identified at the earliest opportunity (Government of Ireland 2019).

In Ireland, the primary postnatal home visit within the first 48 h of discharge from the maternity hospital is the only mandated PHN visit, and it is also considered the most critical visit (Giltenane et al., 2021). The importance of the first postnatal visit and other community‐based interventions has been augmented in the Irish context through the national health policy Sláintecare, and the National Maternity Strategy (Government of Ireland 2016; Barry et al. 2018). Despite the potential benefits of the first postnatal visit for both parents, the PHN service is viewed and delivered as a mother and baby service (Giltenane et al. 2021). The visit is explicitly designed to support women in their transition to motherhood, with a focus on maternal and infant health and well‐being (Giltenane et al. 2022). While the visit is essential for mothers, existing research evidence indicates a lack of engagement with fathers (Leahy‐Warren et al. 2023). This represents a missed opportunity to promote and foster fathers’ capabilities in terms of child welfare and partner support (Philpott and Corcoran 2018). Furthermore, compared to other life stages, the transition to fatherhood and the early years of childrearing are periods when men experience increased risk of adverse mental health outcomes (Watkins et al. 2024).

The risk to father's mental health is compounded by the challenges they encounter when attempting to engage with healthcare services (Venning et al. 2021). There are several reasons why fathers do not engage and seek help for themselves (Schuppan et al. 2019); however, the most commonly reported reason in the literature is the perception that antenatal and early childhood health services are maternal‐centric (Macdonald et al. 2022). Despite this, PHNs consulting with families during the perinatal period are well‐positioned to address the healthcare needs of both parents, with significant benefits for fathers when engagement is meaningful and sustained (Venning et al. 2021). Specific PHN interventions offer valuable ways to support a range of needs not previously considered for fathers during the perinatal period.

The Public Health Intervention Wheel (PHIW), first developed in 2001 by the Minnesota Department of Health, outlines 17 population‐based public health interventions. These interventions are grouped into five color‐coded wedges based on the similarity in function, purpose, and mechanisms of action within public health nursing practice. The red wedge includes interventions such as case management, delegated functions, and case finding, which focus on individualized care and service coordination. The green wedge—comprising health teaching, counseling, and consultation—focuses on communication‐based strategies aimed at knowledge transfer and behavior change. The blue wedge includes surveillance, disease and health event investigation, outreach, and screening, all of which are data‐driven and focused on early identification and monitoring of health issues. The orange wedge groups collaboration, coalition building, and community organizing—interventions that emphasize community empowerment and partnership development. Lastly, the yellow wedge includes advocacy, social marketing, and policy development and enforcement, representing interventions that seek to influence systems and shape policy environments (Keller et al. 2004).

These interventions are applied across three levels of public health nursing practice—individual/family, community, and systems—allowing nurses to address health issues in a comprehensive, multilevel approach. Over the past two decades, the PHIW has been adopted and studied in more than five countries, providing a robust evidence base for guiding public health nursing practice (Schaffer et al. 2022).

The PHIW offers a structured framework relevant to clinical practice, enabling PHNs to identify effective perinatal supports for fathers; however, its application in this context remains underutilized. For example, this author Mulcahy et al. (2016) used a case of Developmental Dysplasia of the Hip to explore the complexities of screening, intervention, and management for a child, family, and the wider population across the three levels of practice. Both authors (L.F.P. and H.M.) published a hypothetical case analysis applying the PHIW to examine PHN practice in falls risk management in older adults across Ireland and Norway. These examples illustrate the range of interventions PHNs implement at all levels and highlights the complexity of practice which can be challenging for novice practitioners to learn. In PHN education, using a case study alongside and the PHIW equips students to identify supports for fathers during the perinatal period.

The use of case studies is an effective educational tool to assist nurses to examine the nuances of clinical practice (Portela Dos Santos et al. 2022). Case‐based enhances academic achievement at both undergraduate and postgraduate levels (Ma and Zhou 2022), and its value in nursing lies in its capacity to teach students the “to think like a nurse” (Dowlman 2022). Peate et al. (2022) in describing the challenges of teaching anatomy and physiology to undergraduate nursing students, suggest that combining clinical reasoning cycles with case studies strengthens students’ ability to relate and apply learning to practice. A qualitative study by Seshan et al. (2021) identified three themes that support effective teaching strategies namely, knowledge development, communication and collaboration, and critical think and problem‐solving. These findings underscore the importance of encouraging students to actively participate in the learning process. A recent scoping review of theories and methods in postgraduate education found that the majority of evidence was medical, descriptive, and originated from the United States (McInerney and Green‐Thompson 2020). This highlights existing gaps for public health nursing educators to address in preparing practitioners for real‐world application. This forms the impetus for developing and analyzing a hypothetical case to illustrate the actual and applicable interventions at individual, family, and community level.

The hypothetical case study outlined below was developed to illustrate the challenges faced by fathers during the postnatal period, particularly the distress and difficulties in coping that may arise when their support needs are overlooked or unmet by healthcare professionals during this life stage. It was designed for use with PHN students and is particularly suited for modules focused on maternal, child, and family health, as well as community and public health nursing. It is intended to be integrated with the PHIW to support critical analysis of how public health interventions can address paternal mental health. Potential instructional strategies include small‐group discussions, guided reflection, intervention mapping using the PHIW, and role‐play to foster interdisciplinary collaboration. Students should also be tasked with identifying gaps in care and proposing evidence‐based interventions aligned with the appropriate levels and functions of PHN practice. This structured, theory‐to‐practice approach enhances learner engagement and promotes deeper understanding of the often‐overlooked area of paternal mental health.

## 2 Hypothetical Case of Paternal Perinatal Mental Health

This hypothetical case is fictional and while influenced by authors’ clinical practice experiences the case cannot be used to identify any individual persons.

### 2.1 Family Context

Parents Mark and Sarah could not have been more excited and prepared for the arrival of their baby Zach. Their pregnancy was planned and eagerly anticipated by both parents. Mark had read every baby book that he could get his hands on, and he had attended all the antenatal appointments with Sarah. Mark was 36 years old, and he felt ready for fatherhood; however, when his baby arrived, the reality of fatherhood was very different to what he had expected.

After the birth, Sarah was struggling with severe low mood and outbursts of anger and frustration which were directed at Mark. Mark felt that he could not do anything right when caring for his baby. Sarah kept criticizing him. He felt that nothing he did made Sarah happy. And then, the baby got croup. Neither of them was getting any sleep and both were stressed and worried. To compound the situation, due to his busy, stressful job, Mark was only able to take 2 days off after the birth of his baby.

Initially Mark seemed to be coping with the challenges he was facing; however, as Sarah's low mood persisted, he began experiencing a decrease in his energy levels and motivation, which was not only impacting his home life, but it was also impacting his performance at work. Sarah was not the only one struggling mentally, silently, Mark was also struggling to hold it all together. Due to her severe low mood, Sarah lacked confidence as a mother, and she felt unable to mind the baby by herself. Physically she was exhausted and unable to do any household duties. Mark felt overwhelmed by the responsibilities of fatherhood.

Getting by on very little sleep and having to watch his wife suffer, took its toll on Mark. He felt sad, lonely, isolated, hopeless, and unsupported. His mood was low, and Mark was struggling with some difficult feelings about his son. He thought that when a baby comes along, that the bond would happen straight away. But that did not happen. He was blaming the baby for what Sarah was going through. He knew that it was wrong to blame his son for the trouble he was seeing his wife go through, but he could not get these thoughts out of his head.

### 2.2 Public Health Nursing Interventions

In the early postnatal period, the PHN Nicola visited Sarah, Mark, and Zach at home. The actual maternal and infant interventions she carried out are outlined below. The PHIW interventions are flagged by bold type. Nicola (PHN) through ongoing **outreach**, **case finding**, and **surveillance** regarding maternal mental health, **screened** Sarah for risk of postnatal depression. A high score on the Edinburgh Postnatal Depression Scale (EPDS) (Cox et al. 1987) meant Sarah met the criteria for **referral** and Nicola **delegated function** through **advocacy, collaboration**, and **coalition‐building** to specialist perinatal mental health services. Adopting a **case management** approach Nicola used **counseling, consultation**, and **health teaching** to plan care for identified sleep, anxiety, and childcare problems. When the PHN Nicola re‐assessed Sarah at **follow‐up** 2 weeks later, in line with her documented care‐plan, Sarah's mood had improved and continued to do so over the next 2 months. Nicola (PHN) also did a full head to toe **assessment** of Zach and performed a newborn bloodspot **screening** test. Zach was jaundiced during the visit and had other concerning features requiring intervention in line with the National Healthy Childhood algorithm. Therefore, Nicola consulted with the maternity hospital and **advocated** for Zach to be **referred** for further assessment.

Unfortunately, Mark did not receive any interventions from the PHN Nicola. Mark did not know there was a name for what he was feeling. He began to think that the negative thoughts and feelings that he was experiencing were a normal part of fatherhood. It did not occur to him to seek help. He was present at the PHN visits so there would have been opportunities to express his needs and thus avail of applicable interventions; however, Mark tried to self‐manage his symptoms by walking and getting sleep when his father‐in‐law came to mind Zach.

It was improvements in Sarah's symptoms that prompted Mark's own recovery. When Mark saw an improvement in his wife's mood, then he started to feel more positive about his family circumstances and this influenced his ability to bond with Zach. It took about 6 months, but fatherhood started to be as Mark had imagined it would be. While Mark suffered during this 6‐month period, it also had a ripple effect on his relationship with his wife and his son. The actual and applicable interventions identified for Mark (father) and Sarah (mother) in this case are listed in Table 1.

**TABLE 1:** Actual and applicable interventions for individuals in the family.

PHN interventions	Mark	Sarah	Zach
Surveillance	app i	√ a i	√ a i
Outreach	app i	√ a i	
Screening	app i	√ a i	√ a i
Case finding	app i	√ a i	
Referral & follow‐up	app i	√ a i	√ a i
Case‐management	app i	√ a i	
Delegated functions	app i	√ a i	
Health teaching	app i	√ a i	
Counseling	app i	√ a i	
Consultation	app i	√ a i	
Collaboration	app i	√ a i	
Coalition—building	app i	√ a i	
Disease/health event investigation	app i	app i	
Community organization	app i	app i	
Advocacy	app i	√ a i	√ a i
Social marketing	app i	app i	
Policy development	app i	app i	

Abbreviations: √ a = actual, app = applicable, i = individual level.

This table illustrates that all actual interventions selected by Nicola the PHN were directed to Sarah and Zach at an individual level. The authors have identified applicable interventions from their knowledge and experience and included them in the table. The hypothetical case will now be used to examine and discuss actual and applicable interventions at all levels (individual, family, and community/systems) for paternal perinatal mental health, using evidence from the literature.

## 3 Discussion

The PHN interventions in the hypothetical case happened at an individual level only. This is not uncommon in practice as highlighted in a recent study which reported that community nursing activities are primarily focused on the individual level, with limited engagement at community and systems levels (Kovacevic et al. 2024). The PHN interventions in the hypothetical case were also maternal and infant focused, which again is typical of PHN practice and reported extensively in the research literature (Giltenane et al. 2021). Despite this, the evidence suggests that fathers would like more formal communication with, and support from the PHN, to augment the informal support they receive within their own network of friends and family (Leahy‐Warren et al. 2023). Considering the variations in fathers' needs and preferences, a trusting relationship with the PHN is essential when expressing their needs and seeking support (Massoudi et al. 2023). If the PHN had considered “family” health, Mark's needs could have been discussed and applicable interventions proposed by the PHN.

The PHN did not use opportunities available to her to discuss with Mark regarding his mental health. He never read an article or saw anything on television in relation to fathers experiencing adverse postnatal mental health. This lack of knowledge among fathers which is not uncommon (Philpott and Corcoran 2018) could be addressed on an individual level through **consultation,** and community level by **social marketing**. During the first and subsequent postnatal visits and appointments, on an individual level, the PHN had an opportunity through **case finding and surveillance,** to **screen** Mark for potential adverse mental health outcomes. She also could have proactively provided **health teaching** regarding the risks to his mental health during the postnatal period. **Health teaching** involves sharing information and experiences through educational activities designed to improve health knowledge, attitudes, behaviors, and skills (Friedman et al. 2011). Such knowledge could have helped Mark discern and distinguish whether he was experiencing the expected challenges of early fatherhood our adverse mental health issues such as stress, anxiety, or depression. If Mark had screened positive for adverse mental health outcomes, he could have been **referred** for further assessment and **follow‐up** through **advocacy**, **collaboration**, and **collation—building** with other healthcare professional in the community such as GPs and mental health services.

Through the integration and implementation of evidence‐based interventions, Mark's distress could have been diminished and his relationship with his partner and infant augmented (Rodrigues et al. 2022) The case highlights the need for PHNs, health services and policy makers to use **outreach** and **social marketing** to increase public awareness at an individual, family, and community/systems levels regarding adverse paternal mental health outcomes. Use of **social marketing** principles results in effective **outreach** messaging. **Outreach** locates populations of interest or populations at risk and provides information about the nature of the concern, what can be done about it, and how to obtain services (Minnesota Department of Health 2019), while **social marketing** is a process that uses marketing principles and techniques to change target audience behaviors to benefit society as well as individuals (Lee and Kotler 2016). The awareness garnered through these activities influences help seeking and health related behaviors among new fathers. Given that there is a lack of public awareness about adverse paternal l mental health (Philpott and Corcoran 2018), and symptoms and effective treatments are not well understood (Khan and Topping 2022), this is likely to also be a specific barrier for men, who are particularly averse to pharmacological treatment for depression (Seidler et al. 2016).

Fatherhood in the postnatal period is affected by social constructions of gender at various levels, including the micro (individual/families), meso (organizations), and macro (policies) levels (Duffey et al. 2020; Hawkes et al. 2021). The lack of attention to gendered realities in **surveillance, case finding, screening, referral and follow‐up, health teaching, counseling, outreach, advocacy,** and **social marketing** by the health service generally and the PHN service specifically—in the context of postnatal mental health unintentionally creates barriers that hinder fathers from recognizing adverse outcomes, seeking help, and considering treatment options (Fisher et al. 2021). Gendered‐based discrimination regarding paternal mental health was made evident with the publication and implementation of the National Model of Care: Specialist Perinatal Mental Health Services in Ireland (Health Service Executive 2016). The model of care was developed to address and implement services and interventions to prevent and address adverse perinatal mental health; however, it focused solely on prevention, screening, and management of maternal health, thus missing an opportunity to include the mental health of the other parent, fathers (Health Service Executive 2016).

While there is a growing body of research literature globally highlighting the impact of paternal postnatal mental health on the overall well‐being of the family, in practice paternal difficulties remain undetected and unmanaged (Leonard et al. 2019). This reflects a lack of inclusion of fathers at policy level (Darwin et al. 2021). Taking the hypothetical case, **developing a policy** would place paternal mental health on the agenda of decision‐makers’, helping to determine the resources needed, and thus resulting in legislation, regulations, decrees, guidelines, and policies (Minnesota Department of Health 2019). **Policy enforcement** would compel those who interact with fathers and their families to observe the guidelines, legislation, regulations, decrees, and policies created in conjunction with policy development (Minnesota Department of Health 2019).

Even though Mark met many healthcare professionals as he navigated his way through the maternity and community services, none of them asked him how he was coping with the transition to fatherhood. Furthermore, he was not informed about potential risk factors or adverse mental health outcomes that he may experience. He wished he was informed as he wanted to find support and be listened to. He also wanted to be **counseled** which would have involved establishing an interpersonal relationship with the PHN with the aim of enhancing and increasing his capacity to coping with his stressful life situation (Keller et al. 2018). **Counseling** is an intervention frequently implemented in conjunction with, or sequentially to **consultation**, which seeks to generate alternative solutions to problems (Keller et al. 2018). Mark was looking for solutions as he tried to manage his symptoms by walking and getting sleep when his mother‐in‐law came to mind Zach. It was improvements in Sarah's symptoms that prompted his own recovery. Sarah's recovery was aided by several public health interventions which were accessible to her. Such interventions were either not available or not implemented with Mark. If Sarah's mental had not improved, how would that have impacted on Mark and Zach both in the short and long term. While Mark fully appreciated that the focus of the PHN service should be on his wife and son, he also now realizes that the postnatal period can be difficult for fathers and help, support and interventions are needed for men too.

## 4 Limitations

Hypothetical case studies, while often used in educating health care practitioners have limitations in terms of oversimplifying the complexity of real life (Yin 2018). However, their value in this paper is that the case served as the starting point for analyzing actual to applicable interventions and discussing the latter in the context of the public health nursing intervention wheel and other evidence‐based literature. Nevertheless, the authors acknowledge the potential bias of their theoretical perspectives as educators (Flyvbjerg 2017) and believe that testing of the case in the educational setting with students is the next logical step.

## 5 Conclusion

This paper was inspired by the clinical and educational experience of the authors. Using scenarios, stories and case studies resonates with public health nursing students and helps them understand the nature and nuances of practice. Integrating a case study with the intervention wheel is not a new phenomenon but there is limited evidence of its utility in educational settings. Paternal perinatal mental health is an area of healthcare which needs greater attention. Thus, choosing a case study and the intervention wheel was deemed an appropriate teaching strategy. It was structured to mirror actual practice where care is focused on mother and infant as frequently witnessed by the authors. Areas where care could have been enhanced through the application of targeted interventions were examined in relation to published literature. The next phase is to design and test the teaching intervention with students.

## Author Contributions

Both authors contributed to the journal article.

## Conflicts of Interest

The authors declare no conflicts of interest.

## Data Availability

Data sharing is not applicable to this article as no new data were created or analyzed in this study.
